# The somatic care of patients with comorbid mental disorders: protocol of a mixed-methods study aiming to identify barriers to and enablers of utilization of somatic healthcare (SoKo)

**DOI:** 10.1186/s12913-023-09525-1

**Published:** 2023-06-07

**Authors:** Sophie E. Groß, Isabell Schellartz, Jürgen Zielasek, Lara Schlomann, Inna Klee, Careen Ritschel, Sandra Engemann, Barbara Steffens, Michaela Jänner, Oliver Funken, Georg Juckel, Euphrosyne Gouzoulis-Mayfrank

**Affiliations:** 1 Rhineland State Council – Institute of Healthcare Research (LVR-IVF), Cologne, Germany; 2 LVR-Clinics Cologne, Cologne, Germany; 3 Medical Faculty, Heinrich-Heine-University, Düsseldorf, Germany; 4 Techniker Krankenkasse North Rhine-Westphalia, German Statutory Health Insurance Company (TK-NRW), Hamburg, Germany; 5 General Practitioners Association North Rhine, North Rhine, Germany; 6 LWL University Hospital, Bochum, Germany; 7 Department of Psychiatry, Psychotherapy and Preventive Medicine, Ruhr University, Bochum, Germany

**Keywords:** Claims data, Comorbidity, General practitioners, Healthcare, Mental disorders, Mixed methods, Patients, Physicians, General practitioners, Specialists, Claims data, Somatic disease

## Abstract

**Background:**

Healthcare for people with somatic and comorbid mental diseases can pose a challenge to the healthcare system. The aim of the SoKo study (the Somatic care of patients with mental Comorbidity) is to assess the current state of care and the facilitators and barriers of somatic care of people with somatic disorders and comorbidity of a mental disorder.

**Methods:**

The study is conducted as a mixed-methods approach and will include (a) descriptive and inferential analysis of secondary claims data of persons insured by a German statutory health insurance company in North Rhine-Westphalia (Techniker Krankenkasse, TK-NRW), (b) qualitative individual interviews and group discussions, and (c) based on (a) and (b), quantitative surveys of both patients and physicians. We intend to analyse a sample of claims data of about 2.6 million persons insured by TK-NRW (group comparisons between TK-NRW insured persons with a diagnosis of a prevalent somatic disease [ICD-10-GM E01–E07, E11, E66, I10–I15, I20–I25, I60–I64] with and without comorbidity of a mental disorder [F00–F99]), in order to assess the uptake of somatic care by people with mental and somatic comorbidity. In addition, primary data from patients with the aforementioned somatic illnesses and a mental comorbidity as well as primary data from physicians (general practitioners and medical specialists) will be collected. The focus here will be on support factors and barriers in the somatic care of people with mental comorbidity.

**Discussion:**

Up to now, there have been no published results of a systematic collection of both secondary and primary data on the utilisation of different care services of somatically ill patients with mental comorbidity for Germany. The present mixed-methods study aims to address this gap.

**Trial registration:**

The trial is registered with the German Clinical Trials Register DRKS: DRKS00030513. The trial was registered on 3rd February 2023.

## Background

Mental disorders are highly prevalent in the general population. In Germany, the 12-month prevalence of mental disorders is about 25–30% of the population [1]. Moreover, comorbidity of mental and somatic diseases is a frequent finding in healthcare data and clinical practice. There are two different ways in which the association of somatic diseases and mental disorders becomes apparent: First, people with somatic disorders have an increased prevalence of comorbid mental illnesses [2, 3]. More specifically, patients with chronic somatic diseases are more likely to develop one of the common mental disorders, such as depression or anxiety disorder, compared to people without somatic diseases [4]. For example, the prevalence of depression is 1.5 to 3 times higher in people with diabetes mellitus [5, 6]. On the other hand, people with several mental illnesses (SMIs), such as depression, bipolar disorder, schizophrenia, and anxiety disorder, have an increased risk of somatic comorbidity [3, 7, 8], especially cardiovascular [3, 9] and metabolic diseases [10]. The increased prevalence of somatic disorders is associated with an increased need for somatic healthcare. For example, the rate of hospitalization for cardiovascular disease is increased by 15% [9]. Somatic-mental comorbidity leads to decreased life expectancy and increased mortality rates [9]. In spite of these adverse health outcomes, studies show that people with SMIs show reduced utilization of healthcare screening and treatment services for somatic diseases such as breast cancer screening in comparison to people without SMIs [11–14]. Furthermore, people with mental disorders tend to receive fewer medical services for cardiovascular disease, diabetes mellitus, and breast cancer than people who are not mentally ill [11, 15]. Furthermore, the comorbidity of mental and somatic illnesses is associated with increased functional limitations, a reduced quality of life, and increased healthcare costs [3, 16]. Especially in SMIs such as schizophrenia, life expectancy is significantly shortened [17]. Potential causes include but are not limited to an unhealthy lifestyle, an accumulation of somatic comorbid diseases, side effects of medication, and inadequate medical care [11, 13, 14, 18, 19]. Taken together, the special healthcare needs of people with comorbid somatic and mental diseases can pose a challenge to the healthcare system [3, 20, 21].

Former studies have focused on the reasons for the increased mortality rates and on the question of how access to health services for people with comorbid mental and somatic disorders can be improved [3, 11–19]. To our knowledge, there are no findings based on the combination of primary and secondary data on the somatic care situation of patients with mental and somatic comorbidity. In the SoKo study (**So**matic care of patients with mental **Co**morbidity), we aim to capture the current uptake of the somatic care of patients with mental comorbidities in Germany and to analyse possible differences of healthcare uptake between the various comorbid mental disorders. Since mental illnesses can be very different in severity and different limitations can accompany them, distinguishing the various manifestations of mental illnesses is indispensable for the analyses. Furthermore, the somatic diseases to be investigated were selected due to their high prevalence, their therapeutic treatability, and their impact in terms of impairments of life expectancy and quality of life. We therefore include diseases of the thyroid gland, arterial hypertension, type 2 diabetes mellitus, obesity, coronary heart disease, stroke, and breast cancer (ICD-10-GM E01–E07, E11, E66, I10–I15, I20–I25, I60–I64). We expect that by choosing these somatic disorders, the implementation of the recommendations for action based on the project results should lead to significant healthcare improvements.

We will analyse statutory health insurance (SHI) claims data of people with mental and the aforementioned somatic diseases to assess healthcare utilization rates in Germany [work package (WP) I]. As little is known about the particular needs of, barriers to, and helpful resources for the somatic care of patients with mental disorders, we will also analyse primary data from interviews/focus groups and questionnaires on the perspectives of both patients (WP II – qualitative data; WP IV – quantitative data) and physicians (WP III – qualitative data; WP V – quantitative data).

By combining results from these different data sources, we aim to develop recommendations to improve access to health services and the quality of healthcare for patients with somatic and comorbid mental illnesses. We anticipate that our research approach will address the following hypotheses:

Primary hypothesis.

1. The somatic care of patients with mental comorbidity is quantitatively and qualitatively different to the somatic care of patients without mental comorbidity.

Secondary hypotheses.

2. Mental and somatic multimorbidity is a barrier to the somatic care of people with mental illnesses.
3. Certain SMIs, such as schizophrenia, are more often a barrier to somatic care than other mental illnesses.
4. Transitional barriers at the interfaces between in Germany traditionally segregated inpatient and outpatient care sectors represent a barrier to the provision of care.
5. Good health literacy is beneficial for the somatic care of patients with mental comorbidity.
6. Good social integration has a positive correlation with somatic care of patients with mental comorbidity.
7. Characteristics of the treating physicians (professional experience, prejudices) can be associated with the somatic care of patients with mental comorbidity.

The primary hypothesis as well as the secondary hypotheses 2–4 will be investigated by analysing the claims data of persons insured with the TK-NRW (WP I). The primary data from interviews and questionnaires is expected to provide relevant content and constructs for the secondary hypotheses 4–7 (WP II – V).

## Methods/design

This study is performed by the LVR-Institute of Healthcare Research (LVR-IVF) in cooperation with the LVR-Clinic Düsseldorf and the Techniker Krankenkasse North Rhine-Westphalia, division of a German Statutory Health Insurance Company (TK-NRW).

The SoKo study includes persons who have already made use of the healthcare system due to common somatic diseases (thyroid gland, arterial hypertension, type 2 diabetes mellitus, obesity, coronary heart disease, stroke, and breast cancer (ICD-10-GM E01–E07, E11, E66, I10–I15, I20–I25, I60–I64, C50)). These somatic diseases include both acute and chronic conditions, which require different types of outpatient, inpatient and rehabilitative care. We will study the somatic care of persons who suffer from at least one of these somatic diseases and compare it to the care of persons who suffer from at least one of these somatic diseases and are also diagnosed with a mental disorder (ICD-10-GM F00–F99). All participants are insured with TK-NRW. TK-NRW will provide anonymised claims data of all members who are diagnosed with at least one of the aforementioned somatic disorders. We will compare the data of persons without a mental disorder and persons who are diagnosed with at least one of the aforementioned somatic disorders and a mental disorder (WP I). In addition, TK-NRW will invite a number of members who are diagnosed with at least one of the aforementionfed somatic illnesses and a mental disorder to participate in the guideline-based interviews and the survey study (WP II and IV). Moreover, we will conduct guideline-based interviews, focus groups, and a survey among general practitioners (GPs) and medical specialists in internal medicine, psychiatry, psychotherapy, and psychosomatics (WP III and V).

The following inclusion criteria will be applied:

### Work package I: claims data of persons with and without mental illnesses

The insured persons’ data are anonymized by TK-NRW and are made available to the LVR-IVF for the analyses.

- Claims data of persons with at least one of the following common somatic diagnoses (only confirmed diagnoses, ICD-10 code (German Modification)): thyroid disease (E01–E07), obesity (E66), chronic ischaemic heart disease (I25), arterial hypertension (I10–I15), type 2 diabetes mellitus (E11), stroke (I60–I64), or breast cancer (C50) *and no diagnosis of a mental disorder* (F00–F99)).
- Billing data of patients with at least one of the aforementioned common somatic diagnoses *and* concurrent healthcare service use (documented in the same billing quarter) due to a *confirmed diagnosis of a mental disorder* (F00–F99).

The number of persons insured with TK-NRW amounts to 2.6 million. According to the Robert Koch-Institute (2017), the prevalence of hypertension in Germany is 30.9% in women and 32.8% in men [22]. According to the Association of Statutory Health Insurance Physicians in the Federal Republic of Germany, insured persons with arterial hypertension according to ICD-10 were the most frequent cases of treatment in medical practices in the North Rhine region in 2021, at 34.1% [23]. We therefore also assume that arterial hypertension is the most common somatic disease among persons insured with TK-NRW, according to the somatic inclusion criteria mentioned above. Based on data for 2015, we estimated in the project proposal that approximately (approx.) at least 40% (n = 1,040,000) of persons insured with TK-NRW have a somatic disease according to the abovementioned inclusion criteria. TK-NRW estimates that 12–15% of the persons insured with TK have a psychiatric diagnosis that requires inpatient or outpatient treatment. Consequently, we estimate that at least 10% of patients who meet the inclusion criteria of the abovementioned somatic diagnosis groups also have a mental disorder (n = 104,000).

The power calculations show that with group sizes of n_1_ = 936,000 (secondary data of persons insured by TK-NRW with somatic diseases according to the inclusion criteria described above and without comorbidity of a mental disorder) and n_2_ = 104,000 (secondary data of persons insured by TK with somatic diseases according to the inclusion criteria described above and with comorbidity of a mental disorder), a power of 90%, an alpha of 0.05, and a sensitivity of |d| = 0.01 (Cohen’s d = 0.01) can be achieved.

Inferential statistical procedures such as T-tests, chi-square tests, and further statistical procedures will be used to compare the groups. In particular, differences between the groups of insured persons with somatic and mental illnesses compared to the group of persons with somatic illnesses only, as well as differences between the subgroups of insured persons with different mental illnesses, will be examined. Regression analyses with clinical and socio-demographic co-factors are planned in order to examine outcomes such as cumulative length of stay in inpatient psychiatric treatment, cross-disciplinary care, diagnostic/therapeutic services, therapy density, and mortality. Based on these analyses, associations between healthcare utilization (e.g. utilization rates of psychiatric-psychotherapeutic and somatic care specialists) and outcomes (e.g. Frequency of disability, rates of retirement due to reduced earning capacity, mortality rates) will be analysed. Statistical tests are performed using the Statistical Package for the Social Sciences (SPSS).

### Work package II: qualitative data of persons with mental illnesses

Access to insured persons who meet the inclusion criteria mentioned under Ib) will be provided by the statutory health insurance company TK-NRW. The mailing addresses are determined of TK-NRW on a key date in the survey period. The selection and postal dispatch is carried out by TK-NRW. We expected to find 104,000 persons who meet the inclusion criteria. Based on the experience of the TK headquarters in previous health services research projects, a response rate of approx. 15% is expected. We estimate that a sample of 500 insured persons will be sufficient to achieve a sample size of about 50 (10%) for individual interviews (telephone and video based).

The insured persons are contacted via TK-NRW in order to increase the willingness to participate. A financial incentive is offered and the relevance of the topic is emphasized in the invitation letter to appeal to the intrinsic motivation of the respondents.

### Work package III: qualitative data of outpatient physicians

To identify factors associated with the somatic care of people with comorbid mental illnesses, focus groups will be conducted with i) medical specialists of psychiatry and psychotherapy, neurology, psychosomatic medicine and psychotherapy, and psychotherapists and ii) and General practitioners (internists and General practitioners) from NRW. According to information from the North Rhine Medical Association (Ärztekammer Nordrhein – ÄKNO) and the Westphalia-Lippe Medical Association (Ärztekammer Westfalen-Lippe – ÄKWL), there are 12,957 general practitioners and specialists with the abovementioned groups in NRW (date of data query at the ÄKWL: 12 November 2019; at the ÄKNO: 19 November 2019). A representative sample of 500 specialists representing approx. 4% of all specialists in NRW will be contacted by post and will be invited to participate in a focus group. A response rate of 10% is expected (approx. 50 participants). The mail addresses will be obtained via internet searches. Factors such as practice location, gender, and specialist discipline will be taken into account when setting up the focus groups (purposeful sampling).

In order to increase the willingness to participate, additional recruitment methods will be used (advertisement at scientific and medical specialist congresses, newsletters of professional associations, personal contacts) and financial incentives will be offered. The relevance of the topic is emphasized in the invitation to participate in order to appeal to the intrinsic motivation of the respondents. In order to ensure transparency for providers a project homepage will be set up.

All individual interviews and focus groups in the work packages II and III are digitally recorded with the consent of the participants, professionally transcribed, pseudonymised, and analysed according to Kuckartz [24]. The aim of the qualitative analyses according to Kuckartz [24] is to develop both inductive and deductive categories that map the data material. In the first step, the category system is formed on the basis of the interview guide and thus follows a deductive category formation. In a second step, new aspects expressed by the interview/focus group’s participants are used for inductive category formation. Since reflexivity of data interpretation and interpersonal consensus-building are central principles of qualitative procedures, several measures are taken for quality assurance: the analysis of the interview material is carried out independently by two researchers (psychologists). This will be followed by consensus-building of the research team (psychologists, social scientist and public health economist). The categories that have been worked out will be discussed in a joint discourse within the research group in order to arrive at an in-depth analysis. To implement the exploratory approach of the primary data collection and the mixed-methods approach, the possible factors modifying the utilization and quality of somatic care of the mentally ill persons identified in work packages II and III will underlie the subsequent development of the questionnaires for work packages IV and V.

### Work package IV: Quantitative data of persons with mental illnesses

Access to insured persons who meet the inclusion criteria mentioned under Ib) will be provided by the statutory health insurance company TK-NRW. The mailing addresses are determined of TK-NRW on a key date in the survey period. The selection and postal dispatch is carried out by TK-NRW. We expected to find 104,000 persons who meet the inclusion criteria. Based on the experience of the TK headquarters from previous health service research projects, a response rate of approx. 15% is expected. We estimate that a sample of 20,000 insured persons will be sufficient to achieve a sample size of about 3,000 (15%) for the anonymous online survey.

The following power calculation refers to independent t-tests. The power calculations show that with group sizes of n_1_ = 104,000 (claims data of TK patients with somatic diseases according to the inclusion criteria described under Ib) and n_2_ = 3,000 (planned number of cases with 15% response rate), a power of 90%, an alpha of 0.05, and a sensitivity of |d| = 0.06 (Cohen’s d = 0.06) can be achieved.

The questionnaire will be developed based on literature research, the results of the qualitative interviews with patients, and the results from the interviews/focus groups with specialists. The questionnaire will furthermore be based on both validated scales and the authors’ own developments based on the qualitative work packages. The barriers to somatic care (secondary hypotheses 2 and 3) can be determined, for example, by the number of outpatient appointments. Transitional barriers at the interfaces between inpatient and outpatient care sectors will be identified in more detail in addition to the secondary claims data and primary data from the providers and insured persons (secondary hypothesis 4). The health literacy of patients can be investigated, for example, by surveying the functional, communicative, and critical health literacy of patients secondary hypothesis 5) [25, 26]. The social inclusion of people with chronic mental illness will be related to healthcare service utilisation as measured by the number of outpatient appointments or medication compliance (secondary hypothesis 5). The questionnaires will be subjected to a pretest before being used with patients in order to gain insight into the processes that occur in the respondents when answering the questions [27].

Difficulties may arise regarding the willingness to participate in the survey. For this reason, the insured persons are contacted via TK-NRW. Furthermore, the number of insured persons contacted is set high enough that an insufficient number of respondents is unlikely. Should this nevertheless be the case, a further survey wave can be initiated. To ensure transparency for insured persons a project homepage will be set up.

### Work package V: quantitative data of outpatient physicians

A representative sample of outpatient physicians and medical specialists described above (WP III) will be invited to participate in a postal survey or, alternatively, an online survey. A representative sample of 6,300, approx. 50% of the physicians in NRW, will be invited to participate in the survey. The addresses will be obtained via internet searches or via the medical associations in NRW (ÄKNO und ÄKWL). We expect a response rate of approx. 15% (n = 945). The following power calculation refers to a one-way between-subjects Analysis of Variance (ANOVA) and shows that with a group size of n = 945 (with a response rate of 15%), a power of 90%, an alpha of 0.05, and a sensitivity of |d| = 0.13 (Cohen’s d = 0.13) can be achieved.

Based on the results of WP II and III, the questionnaire for the treating physicians and specialists will be developed on the basis of literature research, the results of the qualitative interviews with patients, and the results of the interviews/focus groups with physicians in these fields. The basis will be validated scales as well as the study authors’ own developments based on the qualitative work packages. In this survey, among others, specialisation, professional experience, and sociodemographic variables will be related to the self-assessment of patient conversations and (stigmatizing) behaviour (secondary hypothesis 6). The questionnaire will be subjected to a cognitive pretest before being used with caregivers in order to gain insight into the processes that occur in the respondents when answering the questions [27]. Regression analyses are planned to examine sociodemographic and other physician-related factors such as stigmatization, empathy, and working conditions with descriptive and inferential statistical methods.

With regard to the survey of physicians, difficulties may arise with regard to the willingness to participate in the survey. In order to achieve the highest possible response rate the survey will be anonymous.

### Participatory research

The inclusion of patients, physicians, and other scientists in the study is to be practised at several points. From the beginning of the project, an advisory board consisting of affected patients, relatives, outpatient physicians, and scientists was established. During the course of the project, the advisory board will supported the preparation of the structured guideline interviews and the questionnaires. The project results will be critically discussed with the advisory board.

### Adjustments during the project

The project started on 1 July 2020 and will have a duration of four years (ending 30 June 2024). Some modifications became necessary in the course of the project. Their implementation is described below.

#### WP I

The realization of WP I was planned from the third quarter of 2020 to the end of the first quarter of 2022. The data extraction of the TK-NRW claims data (secondary data) was complicated by TK-internal processes and this led to delays in data delivery. The secondary data will be delivered in the second quarter of 2023 and subjected to plausibility checks in the third and fourth quarter of 2023. The delay in WP I had an impact on the subsequent work packages, as it was not possible to use initial results from the routine data analyses when collecting the primary data and designing the structured guidelines and questionnaires.

#### WP II

The conduction of WP II was planned and carried out within the expected timeframe from the first quarter of 2021 to the end of the fourth quarter of 2022. The interviews were conducted via telephone (n = 29) or video conference (n = 17).

#### WP III

WP III was planned and carried out within the expected timeframe from the first quarter of 2021 to the end of the fourth quarter of 2022. Since the recruitment of GPs and their willingness to participate was very low, probably due to the COVID-19 pandemic and the resulting high workload, additional recruitment channels were used. Not only were physicians in private practice contacted by post and invited to participate in focus groups and interviews, but also the study and the importance of the focus group for the study were advertised at congresses and specialist conferences as well as via newsletters of professional associations and personal contacts. Nevertheless, it was not possible to reach the target number of 50 practising doctors in the qualitative interviews and focus groups. Recruitment was stopped when nine practising physicians had been reached. However, it is planned to offer further focus groups at in 2023.

#### WP IV

WP IV was planned and has so far been carried out within the expected timeframe from the first quarter of 2022 to the end of the first quarter of 2024. Since the response rate in the first survey wave by TK-NRW was lower than expected, a second recruitment wave became necessary. 40,000 insured persons were contacted by TK-NRW and invited to participate in the online survey. 2,590 insured persons participated in the online survey.

#### WP V

**Fig. 1:**
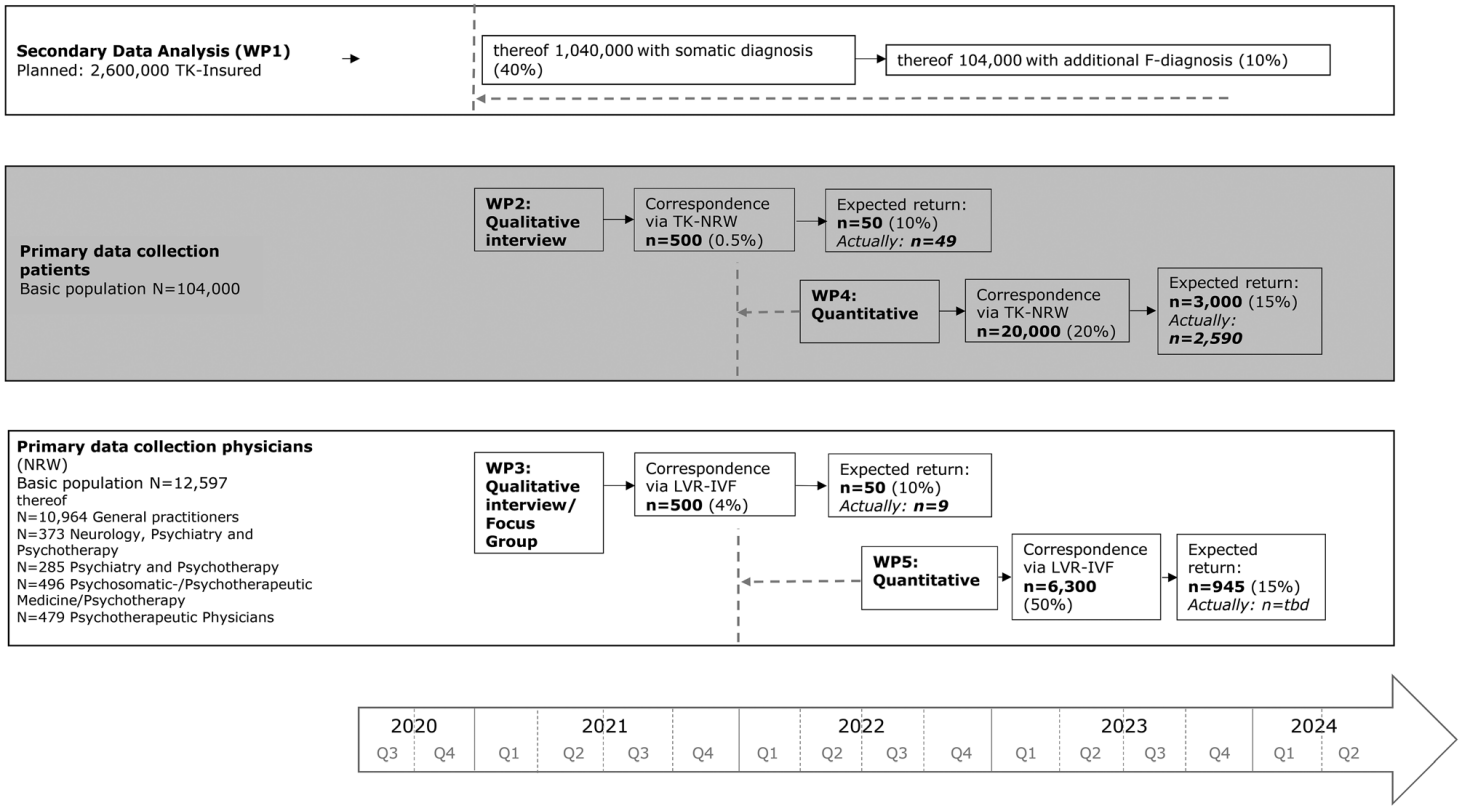
Participants and Timeline

WP V was planned from the first quarter of 2022 to the end of the first quarter of 2024. Due to the persistent high workload in GP practices in the fourth quarter of 2022 in Germany, the recruitment of outpatient physicians for the quantitative survey was postponed to spring 2023. Despite the delayed recruitment of physicians in WP 5, the study team expects to complete WP 5 within the planned timeframe by the end of the first quarter of 2024 (Fig. 1).

## Discussion

Research on the somatic care situation of patients with somatic disorders and comorbid mental disorders compared to patients with somatic disorders without comorbid mental disorders is rare. On the basis of previous studies, it can be assumed that there are differences in the somatic care of patients with mental disorders and patients without mental disorders. Up to now, there are no published results of a systematic collection of primary and secondary data for Germany, especially on the utilization of healthcare services in different sectors (inpatient, outpatient) by somatically ill patients with mental comorbidity. This research gap is to be closed by the present research project. Thus, in addition to the analysis of the primary and secondary healthcare utilization data, the present research project also considers the physicians’ perspective. The strength of this project resides is its mixed methods approach, which brings together the results of qualitative and quantitative methods of primary data collection with the findings of the secondary claims data analyses.

In further studies, the recommendations for action can be implemented and evaluated. The scientific results could also be applied to other relevant care sectors (e.g. hard-to-reach persons) and other indications (e.g. multimorbidity). Another strength of the study is the recruitment of participants via a large SHI company in Germany. This makes it possible for the study team to invite a large number of insured persons and recruit them for the study.

One limitation of our approach to secondary data collection is that the project team is only working with one SHI company from NRW. Unfortunately, a cooperation with other health insurance companies was not possible during the application process, even though this was originally intended by the project team, in order to minimise selection biases considering sociodemographic differences between insure populations in different German SHIs. Due to the cooperation with the largest SHI Company in Germany and also in the federal state of NRW, however, we can minimise the selection bias as far as possible. Another limitation is that we cannot conduct primary data collection nationwide, because through cooperation with TK-NRW and the General Practitioners’ Association North Rhine, primary data can only be collected within NRW. However, it should be noted that NRW is the most populous federal state in Germany and we therefore assume that we will be able to obtain a representative sample of patients and physicians.

The transmission of secondary data was delayed several times due to internal difficulties at TK-NRW, which made adjustments necessary in the work packages, for example the linkage of quantitative data and qualitative perspectives of the mixed-methods approach and adjustments in the timeline. Thus, our strategy for data collection and analyses had to be flexible. One challenge in project implementation was the COVID-19 pandemic. No face-to-face project meetings at the beginning of the study made cooperation difficult in parts. The recruitment difficulties among providers in the qualitative work package (WP III) were severe. Alternative time-consuming recruitment methods had to be used. Nevertheless, the target of fifty participants in interviews or focus groups with healthcare providers could not be reached (WP III). Furthermore, due to the tense situation in the health system in autumn/winter 2022/2023, we will delay the recruitment in WP V of healthcare providers until spring 2023. We hope to achieve a higher response rate by delaying the recruitment of GPs and specialists in private practices until spring 2023.

The study presented here aims to derive recommendations for action based on analyses of the somatic care of patients with comorbid mental disorders, which will provide opportunities to optimize care.

## Data Availability

Not applicable.

## Abbreviations

approx.: approximately
LVR-IVF: Rhineland State Council – Institute of Health Care Research [Landschaftsverband Rheinland – Institut für Versorgungsforschung]
ÄKNO: North Rhine Medical Association [Ärztekammer Nordrhein]
ÄKWL: Westphalia-Lippe Medical Association [Ärztekammer Westfalen-Lippe]
NRW: North Rhine-Westphalia
SHI: statutory health insurance
SPSS: Statistical Package for the Social Sciences
SoKo: The Somatic care of patients with mental Comorbidity
TK: Techniker Krankenkasse, a German statutory health insurance company
TK-NRW: Techniker Krankenkasse Landesvertretung Nordrhein-Westfalen, a German statutory health insurance company in North Rhine-Westphalia
WP: Work Package
